# Assessment of common variability and expression quantitative trait loci for genome-wide associations for progressive supranuclear palsy

**DOI:** 10.1016/j.neurobiolaging.2014.01.010

**Published:** 2014-06

**Authors:** Raffaele Ferrari, Mina Ryten, Roberto Simone, Daniah Trabzuni, Naiya Nicolaou, Geshanthi Hondhamuni, Adaikalavan Ramasamy, Jana Vandrovcova, UK Brain Expression Consortium, Michael E. Weale, Andrew J. Lees, Parastoo Momeni, John Hardy, Rohan de Silva

**Affiliations:** a Laboratory of Neurogenetics, Department of Internal Medicine, Texas Tech University Health Sciences Center, Lubbock, TX, USA; b Reta Lila Weston Institute, UCL Institute of Neurology, London, UK; c Department of Molecular Neuroscience, UCL Institute of Neurology, London, UK; d Department of Genetics, King Faisal Specialist Hospital and Research Centre, Riyadh, Saudi Arabia; e Department of Medical and Molecular Genetics, King's College London, Guy's Hospital, London, UK

**Keywords:** PSP, GWAS, Quantitative trait loci, Sequencing, Haplotype, Linkage disequilibrium, Missense variant

## Abstract

Progressive supranuclear palsy is a rare parkinsonian disorder with characteristic neurofibrillary pathology consisting of hyperphosphorylated tau protein. Common variation defining the microtubule associated protein tau gene (*MAPT*) H1 haplotype strongly contributes to disease risk. A recent genome-wide association study (GWAS) revealed 3 novel risk loci on chromosomes 1, 2, and 3 that primarily implicate *STX6*, *EIF2AK3*, and *MOBP*, respectively. Genetic associations, however, rarely lead to direct identification of the relevant functional allele. More often, they are in linkage disequilibrium with the causative polymorphism(s) that could be a coding change or affect gene expression regulatory motifs. To identify any such changes, we sequenced all coding exons of those genes directly implicated by the associations in progressive supranuclear palsy cases and analyzed regional gene expression data from control brains to identify expression quantitative trait loci within 1 Mb of the risk loci. Although we did not find any coding variants underlying the associations, GWAS-associated single-nucleotide polymorphisms at these loci are in complete linkage disequilibrium with haplotypes that completely overlap with the respective genes. Although implication of *EIF2AK3* and *MOBP* could not be fully assessed, we show that the GWAS single-nucleotide polymorphism rs1411478 (*STX6*) is a strong expression quantitative trait locus with significantly lower expression of *STX6* in white matter in carriers of the risk allele.

## 1 Introduction

Progressive supranuclear palsy (PSP) is a progressive neurodegenerative disorder characterized by early postural instability, supranuclear gaze palsy, and cognitive decline with age at onset typically between 60 and 65 years and 5.8–5.9 years median time from disease onset to death (Burn and Lees, 2002; Williams and Lees, 2009). PSP is a primary tauopathy with widespread tau pathology mainly defined by hyperphosphorylated tau protein, neurofibrillary tangles, neuropil threads and characteristic glial tau inclusions, astrocytic tufts, and oligodendroglial-coiled bodies in the white matter (Dickson et al., 2007).

By virtue of its pathology, it is not surprising that PSP risk has been consistently associated with the H1 haplotype of the tau gene (*MAPT*) (Baker et al., 1999; Conrad et al., 1997; Ezquerra et al., 1999) (for review, see Vandrovcova et al., 2010). While a handful of autosomal dominant PSP cases have been described (Rohrer et al., 2011; Rojo et al., 1999), the majority are sporadic, without evidence of familial clustering. Furthermore some of the *MAPT* mutations that cause familial frontotemporal dementia with parkinsonism linked to chromosome 17 (FTDP-17 and/or FTLD-tau) (Hutton et al., 1998; Spillantini et al., 1998) cause phenotypes resembling PSP (Choumert et al., 2012; Delisle et al., 1999; Morris et al., 2003; Pastor et al., 2001; Poorkaj et al., 2002; Rohrer et al., 2011; Ros et al., 2005b; Rossi et al., 2004; Spina et al., 2008; Stanford et al., 2000).

Elucidation of the possible functional basis of the H1 haplotype association with PSP and corticobasal degeneration (CBD) has led to suggestions of allele-specific differences in transcription and alternative splicing of *MAPT*; both PSP and CBD tau pathology is predominantly 4-repeat tau (4R-tau), that is, consisting of tau protein isoforms with 4 microtubule-binding domains as a result of splicing of *MAPT* exon 10 (Caffrey et al., 2006, 2008; Chambers et al., 1999; Luk et al., 2010; Majounie et al., 2013; Takanashi et al., 2002; Trabzuni et al., 2012). The H1c sub-haplotype within the H1 clade, which drives the association with PSP and CBD (Pittman et al., 2005) was shown to be associated with increased transcription and exon 10 splicing and could thus form the basis of the 4R-tau-dominant pathology (Myers et al., 2007). Other associated loci have been suggested on chromosomes (Chr) 1q31.1 (Ros et al., 2005a) and 11p12 (Melquist et al., 2007) as well as a pV380L polymorphism in parkin (PARK2) (Ros et al., 2008), but these have not been replicated to date.

In 2011, an international consortium published a genome-wide association study (GWAS) that included a large majority of pathologically proven and clinical PSP cases available in Western Europe and the United States (Höglinger et al., 2011). This unequivocally revealed several new associations besides that with *MAPT* (Höglinger et al., 2011). The associated single-nucleotide polymorphisms (SNPs) clustered at 3 different loci on Chr1q25.3, Chr2p11.2, and Chr3p22.1; at the syntaxin 6 (*STX6*), eukaryotic translation initiation factor 2-alpha kinase 3 (*EIF2AK3*), and myelin-associated oligodendrocyte basic protein (*MOBP*) genes, respectively (Höglinger et al., 2011). In addition to the established role of tau, the genes implicated by these associations provide tantalizing suggestions about the cellular pathways and processes that could be affected in PSP, including intracellular vesicular trafficking, central nervous system (CNS) myelination and the endoplasmic reticulum (ER)-mediated cellular response to stress, and abnormally unfolded proteins (Höglinger et al., 2011).

As with all genetic association studies, the next challenge is to determine the functional underpinnings of these associations and how their allelic differences influence risk. In the first place, the strongest associations do not necessarily implicate the nearest gene, but could point to other genes in the vicinity that are in linkage disequilibrium (LD). On rare occasions, the associated SNP(s) are the functional variant(s) by missense change of protein coding sequences or, by influencing important gene regulatory motifs involved in expression or splicing. More frequently, associated SNPs reside within intronic or intergenic regions with the possibility that they act at the RNA level, thus affecting gene expression or they influence the variety of non-coding RNA genes such as micro-RNAs or their targets. Therefore, to delineate the associated region, mapping of LD with the associated SNPs enables us to short list the candidate genes implicated by the associations for more detailed sequence analysis for the identification of high-risk variants that could affect protein or gene function. In addition, the availability of array-based genome-wide gene expression data for various tissues and multiple brain regions (see Trabzuni et al., 2011) now enables expression quantitative trait locus (eQTL) analysis to identify expression and splicing changes that are influenced by individual SNP alleles, be they in cis within the same gene locus or neighboring and distal genes.

The first discovery stage of the PSP GWAS included 141 pathologically confirmed PSP cases from the Queen Square Brain Bank (Höglinger et al., 2011). In this study, we selected 84 of these cases to further investigate the associated loci by direct sequencing of the coding sequences of the implicated genes to identify further causative polymorphisms, including missense changes. Our primary aim was to determine if any coding variability underpinned the reported association. A secondary aim was to identify rarer variants that are key to contributing to disease risk. Furthermore, we carried out analysis of regional genome-wide eQTL analysis in genetically characterized control brains to determine if any of the associated SNPs cause allelic differences in gene expression that might also explain the association with disease.

## 2 Methods

### 2.1 Study population

Eighty-four pathologically confirmed PSP cases were screened in this study, all of which were included in the recent GWAS (Höglinger et al., 2011) and have genome-wide genotype data. They were all white, western European origin, and met modified NINDS possible or probable criteria (Litvan et al., 1996a) and diagnosis was confirmed pathologically using standardized criteria (Dickson et al., 2007; Litvan et al., 1996a, 1996b). All patients were collected under approved protocols followed by informed consent and this work was approved by the Joint Medical Ethics Committee of the National Hospital of Neurology and Neurosurgery, London.

### 2.2 Genetic analysis

Genomic DNA was extracted from dissected samples (100–200 mg) of human postmortem brain tissue using the Qiagen DNeasy Blood & Tissue Kit (Qiagen, Manchester, UK). Genotype data for the 3 GWAS-associated SNPs rs1411478 (Chr1q25.3; P_(joint)_: 2.3 × 10^−10^), rs7571971 (Chr2p11.2; P_(joint)_: 3.2 × 10^−13^), and rs1768208 (Chr3p22.1; P_(joint)_: 1.0 × 10^−16^) were derived from the PSP-GWAS data (Höglinger et al., 2011).

We sequenced all the coding exons and flanking intronic regions of the genes that were within the associated haplotype block (regions of LD of r^2^ > 0.8 with the GWA associated SNPs). These were *STX6* (exons 2–8), trans-membrane protein 1 gene (*MR1*) (exons 2–7), *EIF2AK3* (exons 2–17), and *MOBP* (exons 3–5). Primers for polymerase chain reaction amplification of each exon and immediately flanking introns were designed using Primer3 (http://frodo.wi.mit.edu/primer3/) and are available upon request. Exploratory sequencing of purified polymerase chain reaction amplicons was carried out in a single direction and in both directions to confirm any identified variants. Sanger sequencing was carried out with the Big Dye Terminator kit (ABI, Foster City, CA, USA) following standard protocol as recommended by the manufacturer, and run on a 3730 DNA Analyzer (ABI), followed by analysis with Sequencher 4.9 software (Gene Codes Corporation, Ann Arbor, MI, USA). The sequences of the coding and flanking intronic regions were used to identify disease-associated haplotypes that are in LD with the most strongly associated SNPs from the GWAS. Chr1 (rs1411478 in intron 4 of *STX6*): common coding and noncoding variants in *STX6* and *MR1* were used to build a haplotype spanning ∼70 Kbp. Chr2 (rs7571971 in intron 2 of *EIF2AK3*): common coding and noncoding variants in *EIF2AK3* were used to build a haplotype spanning ∼42.6 Kbp. Chr3 (rs1768208 in intron 2 of *MOBP*): only noncoding variants in *MOBP* could be used to build a haplotype spanning ∼32 Kbp. LD was analyzed using Haploview (www.broadinstitute.org/haploview) (Barrett et al., 2005) and SNAP, the SNP annotation and proxy search program (www.broadinstitute.org/mpg/snap/index.php) (Johnson et al., 2008) for HapMap Release 22 SNP data for CEU (Utah residents with Northern and Western European ancestry from the CEPH collection). The potential damaging effect of any novel missense variants on protein structure and function was examined using PolyPhen-2 software (Adzhubei et al., 2010).

### 2.3 Expression quantitative trait locus analysis

Frozen brain originating from 134 neurologically and neuropathologically control individuals was collected by the Medical Research Council Sudden Death Brain and Tissue Bank, Edinburgh, UK (Millar et al., 2007), and the Sun Health Research Institute, an affiliate of Sun Health Corporation, USA (Beach et al., 2008). From each individual we analyzed up to 10 brain regions: cerebellar cortex (CRBL), frontal cortex (FCTX), hippocampus (HIPP), medulla (specifically inferior olivary nucleus, MEDU), occipital cortex (specifically primary visual cortex, OCTX), putamen (PUTM), substantia nigra (SNIG), thalamus (THAL), temporal cortex (TCTX), and intralobular white matter (WHMT). A detailed description of the samples used in the study, tissue processing and dissection is provided in (Trabzuni et al., 2011). All samples had fully informed consent for retrieval and were authorized for ethically approved scientific investigation (Research Ethics Committee number 10/H0716/3).

Total RNA was isolated from human postmortem brain tissues using the miRNeasy 96 kit (Qiagen). The quality of total RNA was evaluated by the 2100 Bioanalyzer (Agilent, Wokingham, UK) and RNA 6000 Nano Kit (Agilent) before processing with the Ambion WT Expression Kit and Affymetrix GeneChip Whole Transcript Sense Target Labeling Assay, and hybridization to the Affymetrix Exon 1.0 ST Arrays following the manufacturer's protocols (Affymetrix, High Wycombe, UK). Hybridized arrays were scanned on an Affymetrix GeneChip Scanner 3000 7G and visually inspected for hybridization artifacts. Further details regarding RNA isolation, quality control, and processing are reported in (Trabzuni et al., 2011).

All arrays were pre-processed using robust multi-array average normalization (Irizarry et al., 2003) and log_2_ transformation in Affymetrix Power Tools version 1.14–3. In each case, we also calculated the “detection above background” metric. After re-mapping the Affymetrix probe sets onto human genome build 19 (GRCh37) and using Netaffx annotation file Release 31(HuEx-1_0-st-v2 Probeset Annotations), we restricted analysis to 292,000 probe sets which were annotated to have gene names according to NCBI reference sequence build 36 and contained at least 3 uniquely hybridizing probes that were free of common European (frequency > 1%) SNPs or indels (according to the 1000 Genomes Interim Phase v3, March 2012). Gene-level expression was estimated for 26,000 genes by calculating the Winsorized mean (below 10% and above 90%) signal of all probe sets corresponding to each gene. The resulting expression data were adjusted for brain bank, gender, and batch effects in Partek's Genomics Suite v6.6 (Partek Incorporated, USA).

All samples were genotyped on the Illumina Infinium Omni1-Quad BeadChip and on the Immunochip, a custom genotyping array designed for the fine mapping of auto immune disorders (International Parkinson Disease Genomics Consortium et al., 2011). The BeadChips were scanned using an iScan (Illumina, Little Chesterford, UK) with an AutoLoader (Illumina, USA). GenomeStudio v.1.8.X (Illumina) was used for analyzing the data and generating SNP calls.

After standard quality controls (removal of suspected non-European descent individuals, samples with call rate <95% and checks on reported sex status, cryptic relatedness, autosomal heterozygosity rate check, monomorphic SNPs or call rate <95%, no genomic position info or redundant SNPs, *p*-value for deviation from Hardy-Weinberg equilibrium < 0.0001, genotyping call rate <95%, less than 2 heterozygotes present, mismatching alleles with 1000 Genomes project even after allowing for strand), imputation was performed using MaCH (Markov Chain Haplotyping algorithm) software (Li et al., 2009, 2010) and Minimac (http://genome.sph.umich.edu/wiki/Minimac) using the European-Caucasian panel of the 1000 Genomes Project (March 2012: Integrated Phase I haplotype release version 3, based on the 2010-11 data freeze and 2012-03-14 haplotypes). We used the resulting ∼5.88 million SNPs and ∼577,000 indels with good post-imputation quality (Rsq >0.50) and minor allele frequency of at least 5%.

The quantitative trait locus (QTL) analysis was run for each expression profile (either exon-level or gene-level) against every genetic marker (either SNP or indel) in MatrixEQTL (Shabalin, 2012). Subsequent analyses were conducted in R (R Development Core Team, 2011).

## 3 Results

### 3.1 PSP GWAS genes are highly expressed in brain with significant regional variation

We directly sequenced the coding exons and their flanking introns in pathologically confirmed PSP cases and compared minor allele frequencies (MAFs) of known SNPs with data available from 1000 Genomes (http://browser.1000genomes.org/index.html) and dbSNP (http://www.ncbi.nlm.nih.gov/SNP/). Candidate gene sequencing data are detailed in Table 1.

Because most GWAS signals do not appear to act via protein coding changes, we investigated the possibility that they may operate by regulating the expression of proximal and/or distal genes using eQTL analysis. This analysis was performed using paired gene expression and genotyping data generated by the UK Brain Expression Consortium (Ramasamy et al., 2013; Trabzuni et al., 2011, 2012). This data set is based on samples originating from 134 control individuals of European-Caucasian origin. In all cases, there was no history of a neurological disorder and control status was confirmed based on histology and examination by a senior neuropathologist. The individuals sampled had a mean age at death of 58 years, male: female sex ratio of 1:2.8, and the modal cause of death was ischaemic heart disease (44.7%). For each individual, up to 10 anatomic brain regions were sampled (total of 1231 arrays) to provide genome-wide expression data. The brain regions analyzed included those commonly affected in PSP such as the substantia nigra, putamen, hippocampus, and frontal cortex (Schofield et al., 2011; Williams et al., 2007).

**Fig. 1:**
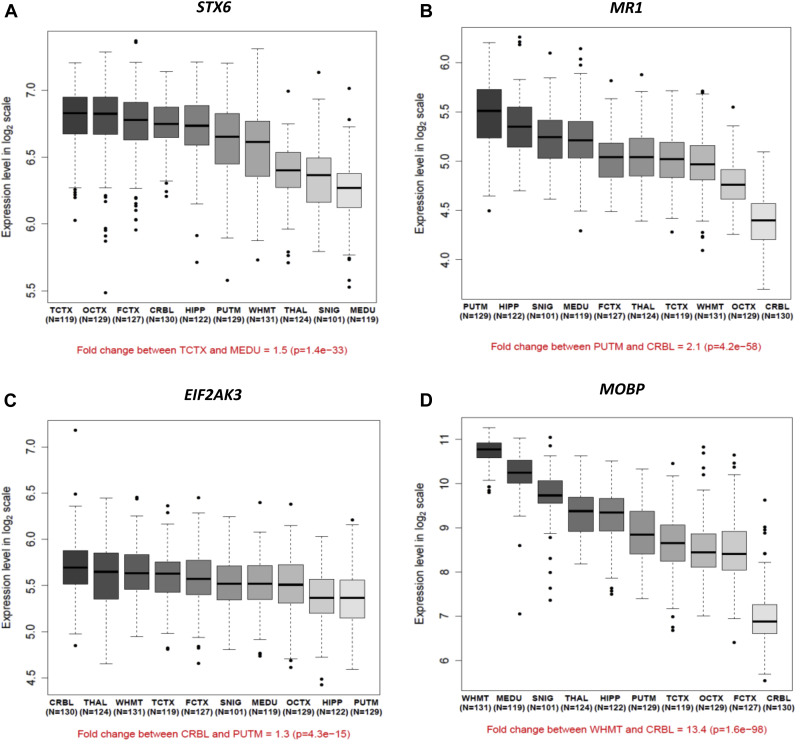
Regional distribution of (A) *STX6*, (B) *MR1*, (C) *EIF2AK3*, and (D) *MOBP* mRNA expression: box plot of mRNA expression levels for 10 brain regions, based on microarray experiments and plotted on a log2 scale (y-axis). These plots show the variation in gene transcript expression across 10 brain regions: the frontal cortex (FCTX, n = 127), temporal cortex (TCTX, n = 119), occipital cortex (specifically primary visual cortex, OCTX, n = 129), hippocampus (HIPP, n = 122), thalamus (THAL, n = 124), cerebellum (CRBL, n = 130), substantia nigra (SNIG, n = 101), putamen (PUTM, n = 129), medulla (specifically inferior olivary nucleus, MEDU, n = 119), and intralobular white matter (WHMT, n = 131). Whiskers extend from the box to 1.5 times the inter-quartile range. Abbreviation: mRNA, messenger RNA.

Of the 26 genetic variants of interest identified in this analysis and the GWAS for PSP, 16 had a MAF of >5% and could be analyzed within our eQTL data set. We checked each genetic variant for evidence of regulation of any gene within 1 mB with particular focus on the effects on *STX6*, *MR1*, *EIF2AK3*, and *MOBP*. With this in mind, we demonstrate firstly the robust expression of these genes in control human brain with significant regional variation (Fig. 1).

### 3.2 Chromosome 1q25.3: *STX6* and MR1

**Fig. 2:**
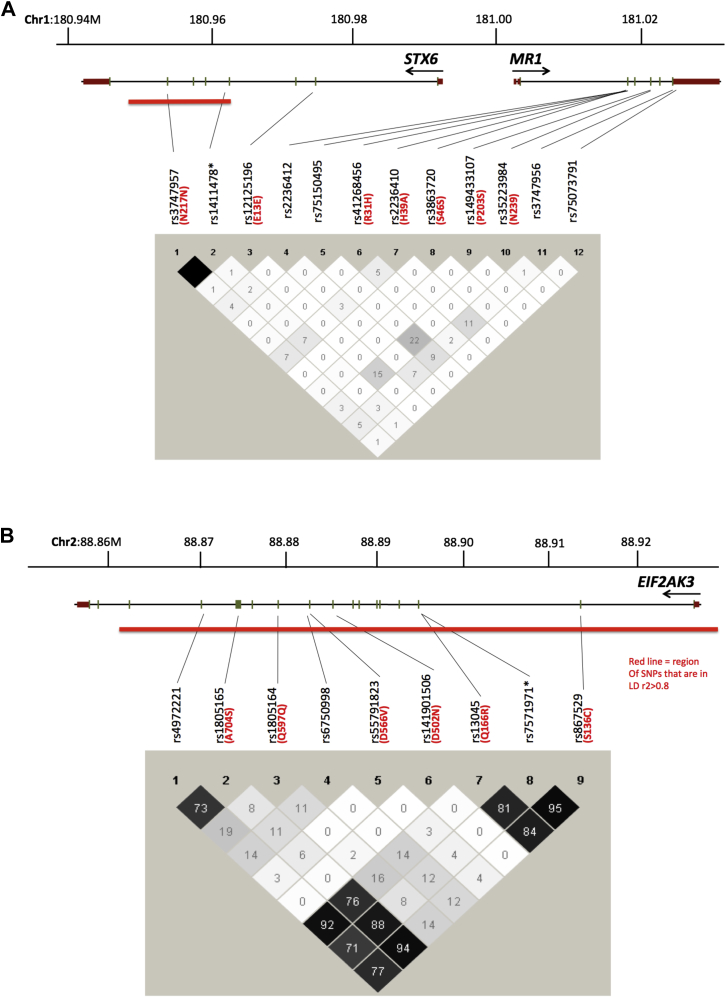
Polymorphisms and linkage disequilibrium at the chromosome 1q25.3 (A) and 2p11.2 loci (B): relative positions of genes and polymorphisms with chromosomal co-ordinates (Mb; genome build hg19/GRCh37) at top. Arrows below gene symbols indicate direction of transcription. In each box the r^2^ value between 2 SNPs is shown with ranges that vary between 0 and 0.99. Any value r^2^ > 0.8 is suggestive of LD. * GWAS associated SNP. Red line indicates linkage disequilibrium plots from CEPH Utah data for northern and/or western European-Caucasian population regions (r^2^ > 0.8) (also see Supplementary Fig. 1). Abbreviations: GWAS, genomewide association study; LD, linkage disequilibrium; SNP, single-nucleotide polymorphism.

Sequencing of *STX6* (Table 1, Fig. 2A) revealed a novel missense variant in exon 8 in a single PSP case causing a Cys_236_Gly substitution (GenBank accession: CAG46671.1) with a PolyPhen-2 score (Adzhubei et al., 2010) of 0.999 (probably damaging; sensitivity: 0.14; specificity: 0.99). The carrier of this mutation did not have any reported history, where available, of PSP or neurodegenerative disorders in direct family members (grandparents, parents, siblings, and children). No information was available for extended family. In addition, we identified 2 known synonymous changes: rs12125196; Glu_13_Glu, and rs3747957; Asn_217_Asn, both with MAFs comparable to the control populations. With reference to the GWAS-associated SNP rs1411478, a cluster of SNPs, including rs3747957 (*STX6* Asn_217_Asn), is in complete LD (r^2^ = 1) forming an LD block spanning the 3′-half of *STX6*, from intron 5 to intron 7 (Fig. 2A, Supplementary Fig. 1A).

In *MR1*, we identified 9 known coding and noncoding variants (Table 1). Five variants are exonic with 3 causing missense changes: rs41268456; Arg_31_His, rs2236410; His_39_Arg and rs149433107; Pro_203_Ser, whereas 2 are silent (rs3863720; Ser_46_Ser and rs35223984; Asn_239_Asn; GenBank accession: CAB77667.1). Only Arg_31_His had a PolyPhen-2 score of 0.996 (probably damaging; sensitivity: 0.55; specificity: 0.98). Four variants are intronic; 2 are proximal to exons (rs75150495, 8 bp upstream from the 5′ end of exon 3, exon 3(-8), and rs3747956, 3 bp downstream from the 3′ junction of exon 7, exon 7(+3)). The latter SNP had a slightly decreased MAF in PSP compared with controls (0.273 vs. 0.494 [1000 Genomes]) but was in line with data from dbSNP (0.273 vs. 0.267–0.302) (Table 1); the MAFs for the other SNPs were comparable with the control populations.

**Fig. 3:**
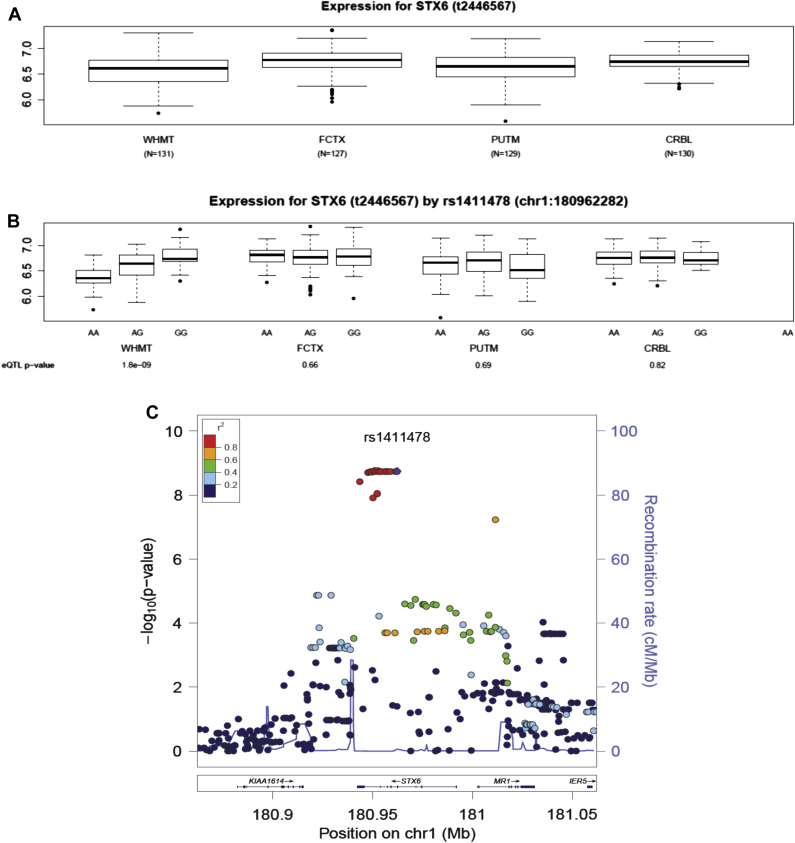
Evidence for a single eQTL signal associated with the PSP risk SNP rs1411478. (A) Box plot showing the distribution of *STX6* gene-level mRNA expression across four brain regions, white matter (WHMT), frontal cortex (FCTX), putamen (PUTM), and cerebellar cortex (CRBL). Whiskers extend from the box to 1.5 times the inter-quartile range. (B) *STX6* gene-level mRNA expression stratified by the genotypes of the PSP risk SNP rs1411478 in WHMT, FCTX, PUTM, and CRBL. The eQTL *p*-values are given on the last x-axis. (C) Regional association plot illustrating the expression quantitative trait loci (eQTL) around the gene *STX6* in WHMT. The PSP risk SNP rs1411478 is shown in purple and the LD measures are with respect to this SNP. Abbreviations: LD, linkage disequilibrium; PSP, progressive supranuclear palsy; SNP, single-nucleotide polymorphism.

Interestingly, the *STX6* rs1411478, is the sole GWA polymorphism in this study that displayed strong evidence as an eQTL. Stratifying messenger RNA (mRNA) levels by genotype of this SNP, we found very strong evidence for the association between the risk allele A and decreased expression of *STX6* (as measured using Affymetrix exon arrays, transcript ID 2446567). This association is evident in white matter (*p* = 1.80 × 10^−9^) but not in other brain regions in the UK Brain Expression Consortium data set (Fig. 3, Supplementary Fig. 2). We also investigated the genomic region around rs1411478 for other SNPs capable of regulating *STX6* expression in white matter and considered all SNPs (genotyped and imputed) within 1 Mb of the transcription start and stop site for this gene (Fig. 3C) without identifying any further strong and significant eQTL signal in this genomic region.

### 3.3 Chromosome 2p11.2: EIF2AK3

We identified several known coding and non-coding variants in *EIF2AK3* (Table 1, Fig. 2B), two of which are intronic; 1 rs6750998, located just 6 bp downstream from the 3′-end of exon 10 (exon 10(+6)). We also identified 6 exonic variants, including 5 that cause missense changes (rs867529; Ser_136_Cys, rs13045; Gln_166_Arg, rs141901506; Asp_502_Asn, rs55791823; Asp_566_Val, rs1805165; Ala_704_Ser) and 1 silent (rs1805164; Gln_597_Gln). Amino acid numbering for EIF2AK3/PERK is with reference to NCBI reference sequence: NP_004827.4. PolyPhen-2 scores predicted all missense variants to be benign besides Asp_566_Val with a score of 0.993 (probably damaging; sensitivity: 0.7; specificity: 0.97). The MAF for rs55791823 (Asp_566_Val) is increased in the PSP cohort compared with 1000 Genomes and dbSNP data for a European-Caucasian normal population (CEU) where the minor allele is very rare (0.016 vs. 0.001 and 0.002, respectively; Table 1). The other *EIF2AK3* SNPs did not differ from the 1000 Genomes or dbSNP data.

Fig. 2B and Supplementary Fig. 2B illustrate LD structure of the associated *EIF2AK3* region. The GWA SNP rs7571971 is in almost complete LD with rs867529; Ser_136_Cys (*D'* = 1, r^2^ = 0.95) and rs13045; Gln_166_Arg (*D'* = 1, r^2^ = 0.81). The SNPs rs7571971 and rs867529 are in almost complete LD with rs1805165; Ala_704_Ser (*D'* = 1, r^2^ = 0.88; *D'* = 1, r^2^ = 0.94, respectively), whereas rs13045 is in almost complete LD with the intronic rs4972221 (*D'* = 1, r^2^ = 0.92), reflecting, however, the exact same LD pattern as in the normal population (www.broadinstitute.org/mpg/snap/index.php).

Gene and exon level expression analysis did not show any significant QTL associations.

### 3.4 Chromosome 3p22.1: *MOBP*

The *MOBP* gene revealed one novel missense mutation (Gln_82_Lys) with a PolyPhen-2 score of benign. The carrier of this mutation did not have any reported history, where available, of PSP or neurodegenerative disorders in direct family members (grandparents, parents, siblings, and children). No information was available for extended family. We also identified 2 known intronic variants (rs2233204 and rs552724). Amino acid numbering for MOBP is with reference to GenBank accession BAA05660.1. The MAFs of the 2 intronic SNPs are overall comparable with 1000 Genomes and European-Caucasian populations (Table 1).

LD analysis did not reveal any LD block within our PSP cohort (Supplementary Fig. 3), whereas the HapMap CEPH Utah data relative to the GWAS associated SNP rs1768208 revealed a cluster of SNPs in high LD (r^2^ > 0.8) spanning a region of ∼25.5 Kb spanning the 5′ half of the gene from, including the first 2 noncoding exons, but excluding the coding exons (Supplementary Fig. 1C). This could implicate 5′ regulatory regions, including the *MOBP* promoter. However, expression analysis did not reveal any gene or exon level QTL associations.

## 4 Discussion

### 4.1 PSP and the *MAPT* locus

Tau has long held pre-eminence in PSP contributing to its definition as a primary tauopathy. This is reinforced by the robust genetic association with the *MAPT* H1 haplotype (Baker et al., 1999; Conrad et al., 1997; Cruchaga et al., 2009; Ezquerra et al., 2011; Pittman et al., 2005), with the recent GWAS showing an odds ratio >5 (P_(joint)_ = 1.5 × 10^−116^) (Höglinger et al., 2011). The identification of a sub-haplotype of H1, namely H1c, and a crucial SNP, rs242557, within a downstream repressor domain in the *MAPT* promoter that defines the H1c haplotype (Pittman et al., 2005), provided evidence that the basis of the association with PSP lies in allelic differences in gene expression (Myers et al., 2007).

### 4.2 Beyond tau

The recent PSP-GWAS revealed a surprising number of robust genome-wide significant associations that implicate novel loci and pathways that contribute to disease pathogenesis (Höglinger et al., 2011). With this work, we used a combination of direct sequencing, and haplotype and eQTL analysis to fine map the loci highlighted by the genome-wide association study (Höglinger et al., 2011) with the aim of better understanding how these loci could contribute to PSP pathogenesis.

### 4.3 Chromosome 1q25.3

At this locus, the associated SNPs are clustered over the *STX6* gene and to a lesser extent, the *MR1*. We demonstrate that the GWAS risk allele rs1411478-A is an eQTL associated with significantly lower expression levels of *STX6* in white matter but not any other brain regions (*p* = 1.80 × 10^−9^) (Fig. 3). In fact, the GWAS SNP, rs1411478 and rs3747957 (Asn_217_Asn) in *STX6* exon 7, are in a group of multiple SNPs that are in complete LD (r^2^ = 1), defining an LD block of ∼14 Kb (Fig. 2A and Supplementary Fig. 1A). This region spans intron 5, exons 6 and 7, and most of the long intron 7. ENCODE tracks (Rosenbloom et al., 2012) for intron 7 indicate a region, relatively conserved in mammals, that is featured by DNAseI hypersensitivity clusters, histone 3 acetylated at lysine 27 (H3K27Ac), and binding of multiple transcription factors from ChIP-seq data. The epigenetic marks are indicative of gene enhancers (Creyghton et al., 2010) that could interact with the distant promoter by the formation of chromatin loops and determine tissue-specific expression programs during development (Ong and Corces, 2011).

It is an intriguing possibility that SNPs in this domain that are in LD with the GWA SNP exert an allele-specific effect on *STX6* expression determined by brain region with the observed eQTL for white matter. Tau pathology has been described in white matter of PSP brains, with overexpression of 4R-tau protein isoforms in the sub-cortical regions and brain stem (Zhukareva et al., 2006), and vacuolation with glial inclusions as a consequence of adjacent gray cellular loss and downstream effects of tau pathology, respectively (Armstrong, 2013). From a functional perspective, STX6 mediates vesicle fusion in diverse transport processes of the exo- and endocytic pathways (Jung et al., 2012) and is mostly involved in late stages of the secretory process (Teng et al., 2001; Wendler and Tooze, 2001). STX6, like the rest of the STX family of proteins, contains a functional soluble N-ethylmaleimide-sensitive factor-attachment protein receptor (SNARE) domain that is responsible for binding to homologue SNARE domains of other SNARE-proteins to direct the transfer of cargo from vesicles to target membranes. Several cell-type specific functions have been mooted (for review, see Jung et al., 2012) including nerve growth factor-dependent neurite outgrowth (Kabayama et al., 2008). It would be intriguing to speculate that the reduction of STX6 expression specifically in white matter associated with PSP risk could be a contributor to the characteristic white matter involvement in PSP, including oligodendroglial tau pathology and/or myelination defects because of impairments in intracellular protein triage and transportation. A possible role of dysmyelination is strongly supported by the involvement of MOBP (see the following details).

Direct sequencing of *STX6* coding exons revealed a single novel missense mutation in *STX6*, Cys_236_Gly, with a Polyphen-2 score of 0.999 (probably damaging) in a solitary PSP case, without any reported family history. Although this mutation seems extremely rare and unlikely explains the association with PSP at this locus in our cohort, functional studies are warranted to assess its pathogenicity and whether it could affect and impair STX6 function.

*MR1* screening identified one missense change rs41268456; Arg_31_His in the conserved extracellular α1 ligand-binding domain (Abos et al., 2011) and has a PolyPhen score of 0.996 (probably damaging) but is very rare with a single heterozygote in the PSP cases and comparable MAFs for PSP (0.007) and 1000 Genomes controls (0.004). Because of the lack of LD between the GWA SNP, rs1411478 and the variants we identified in *MR1* and the absence of any significant gene-specific QTLs, it is less plausible that *MR1* is responsible for the observed association of this locus with PSP.

### 4.4 Chromosome 2p11.2

*EIF2AK3* codes for the eukaryotic translation initiation factor 2-alpha kinase 3, also known as protein kinase R-like endoplasmic reticulum kinase (PERK), a type-I transmembrane protein in the ER and plays a key role in the unfolded protein response (UPR). UPR is initiated during ER stress when accumulation of unfolded protein in the ER lumen exceeds the capacity of ER-associated degradation (Imrie and Sadler, 2012; Mori, 2009; Rutkowski and Kaufman, 2004). There is mounting evidence that dysregulation of UPR or chronic ER stress is a fundamental process in neurodegenerative disorders associated with insoluble protein aggregate formation (Matus et al., 2011) and UPR impairment plays a critical role in the development of tauopathies in that activated UPR enhances tau phosphorylation tau contributing to the formation of neurofibrillary tangles (Fu et al., 2010; Nijholt et al., 2012; Park et al., 2009; Resende et al., 2008).

UPR is activated in PSP brain regions most affected by tau pathology, as well as in Alzheimer's disease and the tau variants of FTLD (Hoozemans et al., 2009; Nijholt et al., 2012; Stutzbach et al., 2013). Activated phospho-PERK colocalizes in neurons with pathologically phosphorylated tau with suggestion that UPR activation may be an early event in tau pathogenesis (Hoozemans et al., 2009; Stutzbach et al., 2013).

More recently, it was shown that specific inhibition of PERK kinase activity prevented disease development in a mouse model of clinical prion disease (Moreno et al., 2013). This neuroprotective mechanism occurs independently and downstream to the process of prion propagation and aggregation (Moreno et al., 2013), and noting the activation of UPR in the tauopathies, it is possible that PERK inhibition could present as a therapeutic target in neurodegenerative tauopathies and proteopathies in general.

We identified rs5579182 (Asp_566_Val), affecting a conserved residue in the cytoplasmic domain, next to the kinase domain of PERK, a heterozygous change in 2 of 61 cases and was predicted being probably damaging. The MAF of this variant has a higher frequency compared with 1000 Genomes and European-Caucasian population data from dbSNP (0.016 vs. 0.001 and 0.002; Table 1) but, for statistical comparison, needs to be studied in larger cohorts.

The GWAS SNP rs7571971 belongs to a region of LD (r^2^ > 0.8) extending to ∼72 kb that spans most of the *EIF2AK3* gene (Stutzbach et al., 2013) (Supplementary Fig. 1B). This includes the coding SNPs rs867529; Ser_136_Cys, rs13045; Gln_166_Arg and rs1805165; Ala_704_Ser and the intronic, rs4972221 resulting in the functional coding haplotypes: A: Ser_136_-Arg_166_-Ser_704_; B: Cys_136_-Gln_166_-Ala_704_, and D: Ser_136_-Gln_166_-Ser_704_ (Liu et al., 2012). Haplotypes A (0.646) and B (0.294) are common in European-Caucasians (CEU) whereas haplotype D (0.029) is rare and haplotype B was shown to be associated with lower bone mineral density (Liu et al., 2012) (Table 2). More recently, the GWAS SNP rs7571971 associated minor T-allele was shown to be in complete LD with haplotype B, whereas the major C-allele did segregate with haplotypes A and D (Table 2) in a cohort of about ∼1000 of the pathologically confirmed PSP cases including those in this study (Stutzbach et al., 2013). Although our study population was relatively small (n = 84), implying a decrease in the power of association analysis, when comparing the frequencies of the sub-haplotypes associated with *EIF2AK3*, the results for our cohort were in line with those of Stutzbach et al. (2013), especially in the case of haplotype B (0.287 vs. 0.288) (Table 2), which was suggested bearing risk for PSP because of increased activity of EIF2AK3 (Liu et al., 2012).

The lack of significant gene or exon level QTLs for *EIF2AK3* is supported by Liu et al. (2012), who showed no haplotype-specific *EIF2AK3* mRNA differences in lymphoblastoid cell lines. However, cell lines with haplotype B showed increased sensitivity to thapsigargin-induced ER-stress as measured by increase in phosphorylated eIF2α levels (Liu et al., 2012), suggesting different functionality of the coding haplotypes. This could be because of impaired activity of the associated haplotype B compared with the major allele, haplotype A (Ser_136_-Arg_166_-Ser_704_) that is completely conserved in mammals. The Ser_136_ and Arg_166_ residues reside in the lumenal domain of the ER and could thus have a role in recognizing the unfolded proteins.

### 4.5 Chromosome 3p22.1

*MOBP* encodes the small myelin-associated oligodendrocyte basic protein that is the third most abundant protein in CNS myelin. Like myelin basic protein, it is small, basic, and localized to compact myelin, preferentially in the major dense lines with a role in compaction and normal arrangement of the radial component of myelin (Yamamoto et al., 1994, 1999; Yoshikawa, 2001). Rat *Mobp* is subject to complex alternative splicing, including the use of alternate exons and 5′ and 3′ splice sites resulting in at least 5 isoforms, all sharing a 68-residue amino terminus (McCallion et al., 1999; Montague et al., 1998) that are targeted to different subcellular organelles including the mitochondria (Montague et al., 2005). Multiple mRNAs and expressed sequence tags suggest similar complexity of human *MOBP*, with alternate splicing and alternate exon and splice site usage. Alternative splicing of coding exons results in 2 protein isoforms of 181 and 83 residues, both sharing the 69-residue amino-terminal domain, whereas the larger isoform has an unusual proline-rich carboxy-terminal domain with 4 perfect PRSPPRSERQ repeats (Yamamoto et al., 1994) with a possible structural role in stabilizing the multilayered structure of the myelin sheath in the CNS (Yamamoto et al., 1994). More recent work implicates MOBP with other myelin proteins as primary antigens in the autoimmune response in multiple sclerosis (Kaushansky et al., 2010).

Our genetic screening revealed a novel missense variant, Gln_82_Lys (1/63, heterozygous change) in the proline-rich domain of the larger isoform. It is predicted to be benign. We also identified 2 common intronic variants (Table 1). Relative to the GWAS SNP, rs1768208, several SNPs spanning the 5′-half of the gene, including the first non-coding exon, are in LD (r^2^ > 0.84) (Supplementary Fig. 1C) but, neither of the noncoding SNPs we identified are in this LD block. Expression analysis both at the transcription and splicing level did not show and significant QTLs. Our data thus did not show evidence implicating any functional underpinnings of the association of *MOBP* with PSP. However, oligodendroglial tau pathology in the form of coiled bodies is characteristic for PSP. The latter occur with tau aggregates appearing as thread-like processes in oligodendrocytic processes ensheathing damaged axons. Some of these processes in white matter are found within both the inner and outer surfaces of myelin sheaths, corresponding to the inner and outer loops of oligodendrocytes (Dickson, 1999; Ikeda et al., 1994; Komori, 1999). This suggests dysfunction or a reactive response of the oligodendrocytes that could be linked to *MOBP* via myelination defects.

## 5 Conclusion

The recent GWAS confirmed the robust association of the *MAPT* locus with PSP with an odds ratio around 5.4 (Höglinger et al., 2011). Several studies have shown allele-specific differences in *MAPT* transcription and splicing with FTDP-17/FTLD-tau mutations at exon 10, and it is clear that imbalances in the 4R-tau/3R-tau ratio can directly lead to neurodegeneration.

The novel loci provide tantalizing glimpses of crucial cellular pathways, including intracellular vesicular transport and fusion to host organelles, the cell's response to accumulation of abnormally unfolded or misfolded proteins and CNS myelination that, if defective, could lead to PSP pathogenesis. Although we did not identify any overt coding or regulatory polymorphisms that explain the associations, the genome-wide associated SNPs are in regions of strong LD that completely overlap with the *STX6*, *EIF2AK3*, and *MOBP* genes. This reiterates these genes as the carriers of the associated risk and suggests that the functional underpinnings of these associations could be in intronic, regulatory regions, including transcriptional enhancers or non-coding genes associated with these loci. For example, the *STX6* risk allele correlates with transcription in white matter and could have a bearing on white matter degeneration and tau pathology. Similarly, the *EIF2AK3* features three coding haplotypes with association with PSP suggesting functional differences in the protein variants (Liu et al., 2012; Stutzbach et al., 2013) whereby, UPR activation acts not as a protective response but rather increases risk of PSP. Not least, *MOBP* is mainly expressed in white matter and there is evidence of white matter involvement in PSP as well as tau pathology affecting white matter. Further studies are needed to confirm, support, and expand these possibilities.

## Disclosure statement

The authors declare no actual or potential conflicts of interest. The funders had no role in study design, data collection and analysis, decision to publish or preparation of the manuscript.

## Figures and Tables

**Table 1:** Summary of the sequencing results for *STX6* and *MR1* (1q25.3), *EIF2AK3* (2p11.2), and *MOBP* (3p22.1). Each known SNP is listed in the “rs number” column. Novel changes are indicated in bold. Minor allele frequencies (MAFs) are shown and compared with those of the normal population obtained from 1000 genomes (http://browser.1000genomes.org/index.html) and dbSNP (http://www.ncbi.nlm.nih.gov/SNP/)

Gene	Exon	Intron	rs number	AA change	Alleles and/or frequency			Minor allele	MAF		
									Our samples	1000 genomes	Caucasian and/or European (dbSNP)
*STX6*	2		rs12125196	Glu13Glu	GG (58/60)	GA (2/60)	AA (0/60)	A	0.017	0.011	0.017–0.025
		4	ars1411478	—	GG (21/82)	GA (44/82)	AA (17/82)	A	0.475	0.426	0.375–0.473–0.483
	7		rs3747957	Asn217Asn	TT (14/62)	TC (33/62)	CC (15/62)	T	0.492	0.427	0.473–0.483
	8		**Novel**	**Cys236Gly**	**TT (57/58)**	**TG (1/58)**	**GG (0/58)**	**G**	**0.009**	**NA**	**NA**
*MR1*		2	rs2236412	—	TT (64/70)	TC (5/70)	CC (1/70)	C	0.050	0.050	NA
			rs75150495	—	CC (63/70)	CG (4/70)	GG (3/70)	G	0.070	NA	NA
	3		rs41268456	Arg31His	GG (69/70)	GA (1/70)	AA (0/70)	A	0.007	0.004	NA
			rs2236410	His39Ala	AA (54/70)	AG (16/70)	GG (0/70)	G	0.114	0.206	0.150
			rs3863720	Ser46Ser	GG (69/70)	GA (0/70)	AA (1/70)	A	0.014	0.017	0.042
	5		rs149433107	Pro203Ser	CC (78/79)	CT (1/79)	TT (0/79)	T	0.006	0.001	NA
			rs35223984	Asn239Asn	CC (74/78)	CT (4/78)	TT (0/78)	T	0.026	0.014	0.033
		7	rs3747956	—	GG (35/64)	GA (23/64)	AA (6/64)	A	0.273	0.494	0.267–0.302
			rs75073791	—	GG (62/64)	GA (2/64)	AA (0/64)	A	0.016	0.011	0.050
*EIF2AK3*	2		rs867529	Ser136Cys	CC (31/63)	CG (28/63)	GG (4/63)	G	0.286	0.288	0.267–0.292
		2	ars7571971	—	CC (36/80)	CT (40/80)	TT (4/80)	T	0.300	0.287	0.267–0.317
	3		rs13045	Gln166Arg	AA (6/59)	AG (25/59)	GG (28/59)	A	0.313	0.347	0.300–0.343
	9		rs141901506	Asp502Asn	GG (57/58)	GA (1/58)	AA (0/58)	A	0.009	NA	NA
	10		rs55791823	Asp566Val	AA (59/61)	AT (2/61)	TT (0/61)	T	0.016	0.001	0.002
		10	rs6750998	—	AA (39/59)	AT (15/59)	TT (5/59)	T	0.212	0.196	0.242–0.258
	11		rs1805164	Gln597Gln	AA (33/65)	AG (26/65)	GG (6/65)	G	0.292	0.293	0.270–0.325–0.350
	13		rs1805165	Ala704Ser	GG (3/43)	GT (17/43)	TT (23/43)	G	0.267	0.287	0.267–0.274–0.280–0.292
		13	rs4972221	—	AA (30/64)	AT (25/64)	TT (9/64)	T	0.336	0.347	0.300–0.317
*MOBP*		2	ars1768208	—	CC (32/82)	CT (43/82)	TT (7/82)	T	0.347	0.349	0.292–0.295–0.317
	3		**Novel**	**Gln82Lys**	**CC (63/64)**	**CA (1/64)**	**AA (0/64)**	**A**	**0.008**	**NA**	**NA**
		3	rs2233204	—	CC (30/68)	CT (27/68)	TT (11/68)	T	0.360	0.196	0.283–0.317–0.327
		4	rs552724	—	CC (54/69)	CT (8/69)	TT (6/69)	T	0.145	0.210	0.183–0.195

Key: GWAS, genomewide association study; PSP, progressive supranuclear palsy; SNPs, single-nucleotide polymorphisms.

a PSP-GWAS associated SNPs.

**Table 2:** Functional haplotypes associated with the *EIF2AK3* locus (2p11.2). Haplotype B has been associated with activation of the UPR processes (Liu et al., 2012) and increased risk of developing PSP (Stutzbach et al., 2013). The frequency of the B haplotype (bold) in our PSP cohort overlaps with that identified on other PSP cohorts (Stutzbach et al., 2013)

Haplotype	SNP and/or amino acid change				Haplotype frequency per study			
	rs867529/(Ser136Cys)	rs7571971^a^	rs13045/(Gln166Arg)	rs1805165/(Ala704Ser)	HapMap CEU	Liu et al	Stutzbach et al	Our study
A	C/(Ser)	C	G/(Arg)	T/(Ser)	0.646	0.676	0.645	0.661
**B**	**G/(Cys)**	**T**	**A/(Gln)**	**G/(Ala)**	0.294	0.311	0.288	0.287
C	C/(Ser)	C	G/(Arg)	G/(Ala)	/	0.013	0.001	0.011
D	C/(Ser)	C	A/(Gln)	T/(Ser)	0.029/0.016	/	0.061	0.033

Key: GWAS, genomewide association study; PSP, progressive supranuclear palsy; SNP, single-nucleotide polymorphism; UPR, unfolded protein response.

a PSP-GWAS associated SNP.
