# Corrigendum to “Assessment of common variability and expression quantitative trait loci for genome-wide associations for progressive supranuclear palsy.” [Neurobiol. Aging 35 (2014) 1514.e1–1514.e12]

**DOI:** 10.1016/j.neurobiolaging.2015.10.005

**Published:** 2015-11

**Authors:** Raffaele Ferrari, Mina Ryten, Roberto Simone, Daniah Trabzuni, Nayia Nicolaou, Geshanthi Hondhamuni, Adaikalavan Ramasamy, Jana Vandrovcova, UK Brain Expression Consortium, Michael E. Weale, Andrew J. Lees, Parastoo Momeni, John Hardy, Rohan de Silva

In the above-mentioned article, the fifth author’s given name should read as ‘Nayia’ instead of ‘Naiya’. The corrected spelling is shown above.

