# Supplementary figures and images for: Assessment of common variability and expression quantitative trait loci for genome-wide associations for progressive supranuclear palsy
Source: Neurobiol Aging. 2014 Jun;35(6):1514.e1–1514.e12. doi: 10.1016/j.neurobiolaging.2014.01.010 (PMC4104112; doi:10.1016/j.neurobiolaging.2014.01.010)

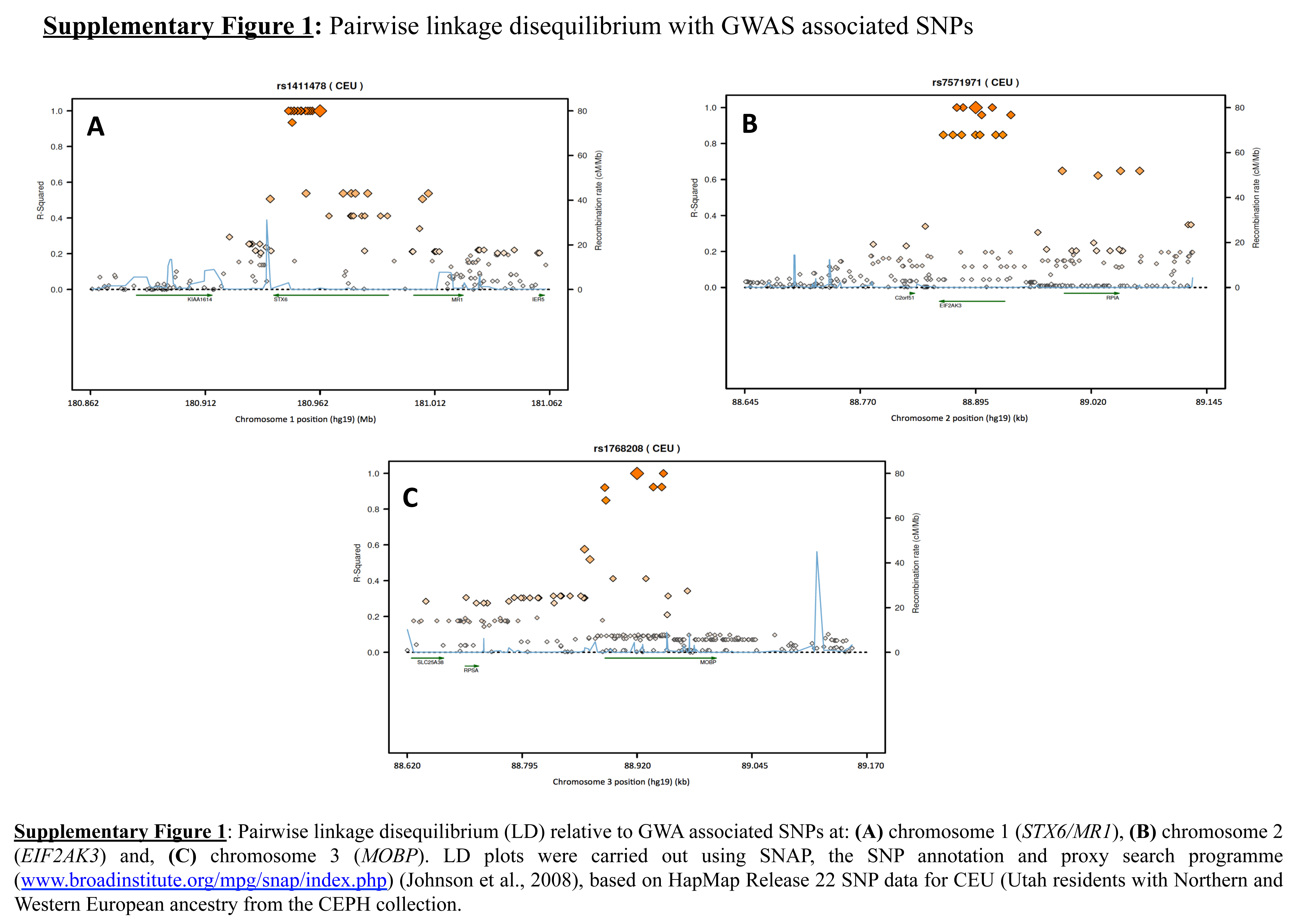


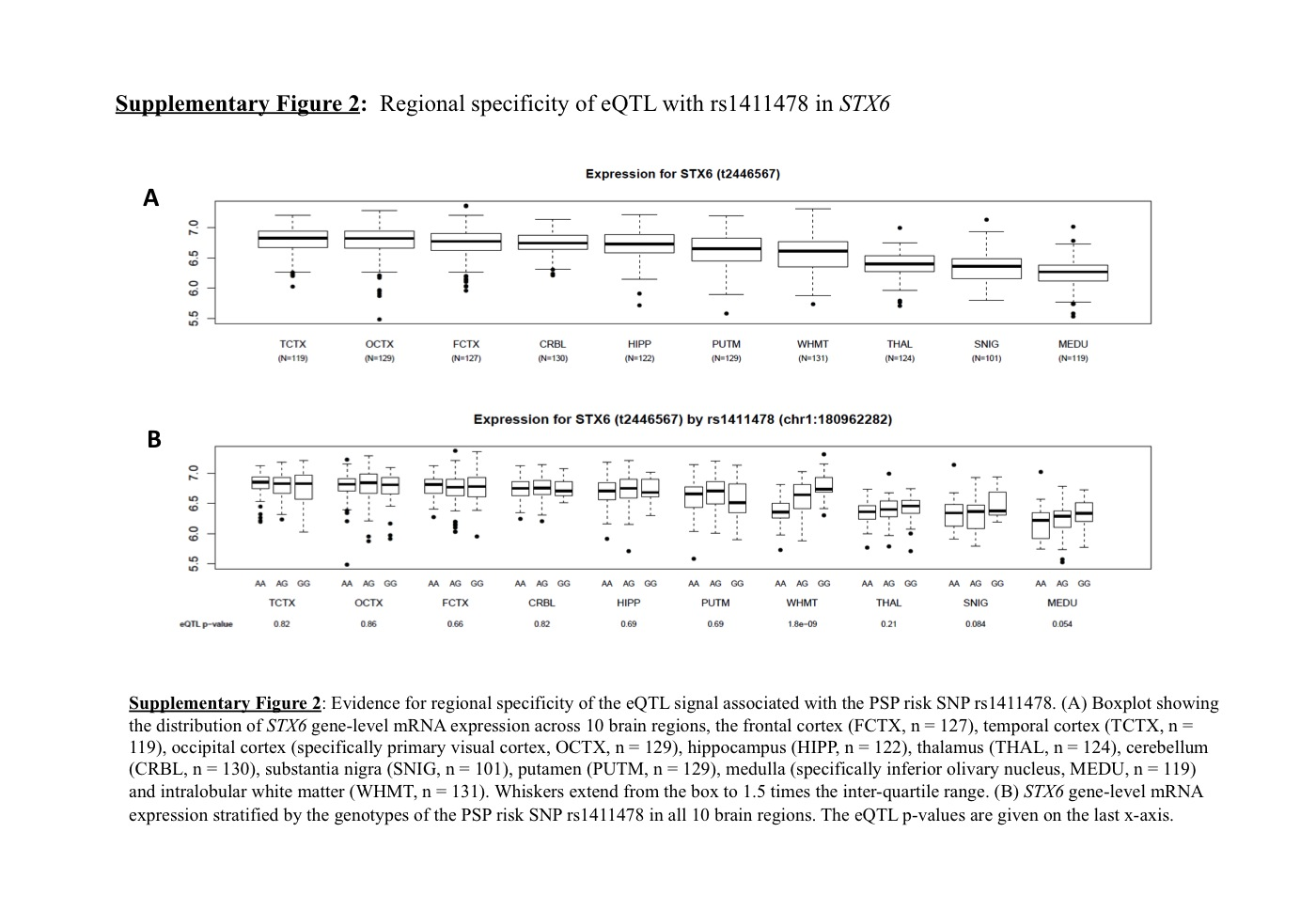


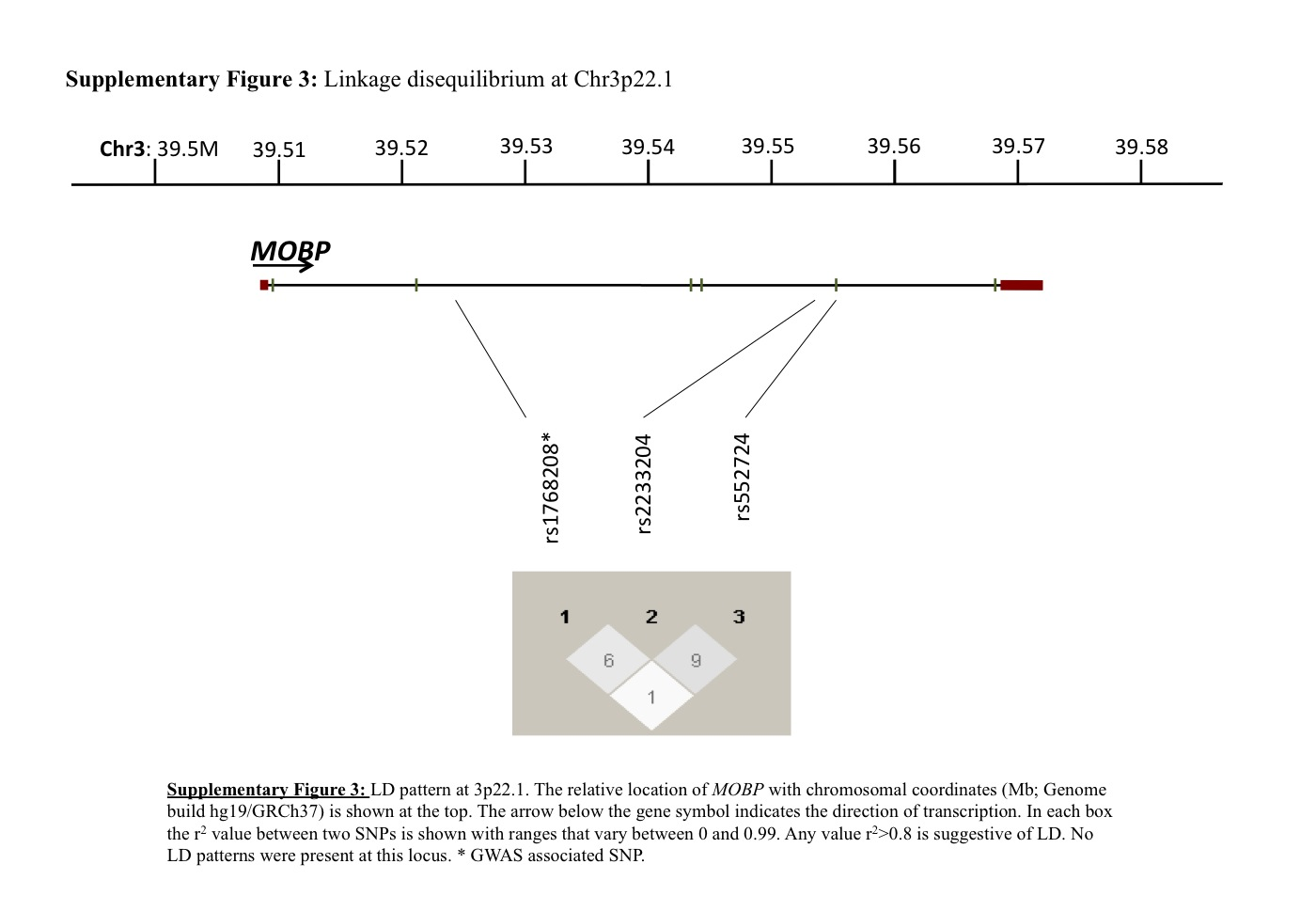

Supplement: Supplementary Figs.1–3 [file mmc1.docx]
